# Mercury in the Diatoms of Various Ecological Formations

**DOI:** 10.1007/s11270-018-3814-1

**Published:** 2018-05-11

**Authors:** Magdalena Bełdowska, Aleksandra Zgrundo, Justyna Kobos

**Affiliations:** Institute of Oceanography, University of Gdańsk, Av. Marszałka Piłsudskiego 46, 81-378 Gdynia, Poland

**Keywords:** Hg, Diatoms, Epilithon, Epiphyton, Phytoplankton, High-profile guild

## Abstract

Mercury is a neurotoxin, its main source in the human organism being fish and seafood. The first level in the marine food web is formed of planktonic and benthic photosynthetic microorganisms, which form a biofilm on the surface of the hard bottom (epilithon) or plants (epiphyton). They are carriers of nutritional as well as toxic substances and pass these on to subsequent levels of the trophic web. Their biomass is often dominated by diatoms. This was the basis for the presented study into Hg accumulation in epilithic, epiphytic and planktonic diatoms, which was carried out in 2012–2013 in the coastal zone of the Puck Lagoon and the Gulf of Gdańsk (southern Baltic). In this coastal area, both micro- and macroorganisms develop particularly intensively. The collected results indicate an increase in Hg concentration in the biofilm during the warm season which, with the lengthening of the vegetative period due to global warming in recent years, is of great significance. As a consequence, the annual mercury load entering the trophic web is larger in comparison with a year in which there is a long, cold winter. An important parameter influencing the accumulation of Hg was the function of those organisms from the biofilm-forming communities. In this case, the highest concentrations of Hg were measured in organisms forming high-profile guilds.

**Electronic supplementary material:**

The online version of this article (10.1007/s11270-018-3814-1) contains supplementary material, which is available to authorized users.

## Introduction

Mercury (Hg) is a highly toxic element for all living organisms—both for bacteria and fungi, as well as for animals. Humans should avoid it due mainly to its neurotoxic properties. It causes among other things autism, Alzheimer’s, depression and schizophrenia (Bose-O’Reilly et al. 2010; Carocci et al. 2014). This metal is also a causative agent for nephrological, motor, immunological, reproductive, cardiac and even genetic problems (Gibb and O’Leary 2014). The main route of Hg penetration into the human body is the consumption of fish and seafood, so great attention is paid to Hg research in the trophic networks of the seas and oceans. Hg undergoes biomagnification; therefore, essential for overall impact is the load of it in the first link of trophic web. The basis of most trophic chains is primary producers or organisms capable of photosynthesis. These organisms belong to different taxonomic groups, and what connects them is their function in the ecosystem—the processing of inorganic compounds into organic by means of solar energy. Hence, they are an important dietary component for organisms on higher trophic levels. In water, primary producers take two forms: phytoplankton, associated with the water body, and phytobenthos, associated with the bottom. Phytoplankton, which contains microscopic cyanobacteria and algae, is a food source not only for pelagic creatures (zooplankton, herbivorous fish or cetaceans), but also for benthic organisms (such as molluscs). Primary production of phytoplankton in oceans is estimated at 30–60% of total primary production on Earth (Cloern et al. 2014) and exceeds the production of tropical forests. Contemporary studies conducted in the Puck Lagoon emphasise that herbivorous fish and benthic organisms tend to feed more on the microscopic organisms, which form biofilm on plants (epiphyton) or other submerged elements (epilithon), than on macrophytobenthos (Jankowska et al. 2016).

In recent years, mercury has been widely used in many branches of industry. In the second part of the XX century, men realised how toxic and dangerous this metal is. In recent decades, the use and emission of mercury has been reduced in many countries; however, its concentration in the environment has not decreased in proportion (HELCOM 2010). This is due, among other things, to the fact that Hg compounds are generally very reactive and can be re-circulated under the influence of changing environmental conditions such as salinity, oxygenation and water temperature. The intense precipitation plays a major role in the delivery of Hg to marine ecosystems via atmosphere and rivers (Saniewska et al. 2014a; b; c). At present, in many parts of the world, climate warming is being observed. In the southern Baltic region, the number of days warmer than 5 °C has increased, while the number of days colder than 0 °C has decreased (Kożuchowski 2009; IMGW PIB 2016; HELCOM 2013). According to the Climate Monitoring Bulletin of Poland, since 1988, there have been more and more years classified as being above the thermal norm—as many as 24 times in 28 years, including four ‘extremely warm’ years and seven ‘anomalously warm’ years (IMGW PIB 2016). Temperature changes affect the circulation of mercury in the marine environment, especially in estuaries or small bays (Bełdowska et al. 2013; 2016). The rise in temperature often leads to a lack of freezing over in winter and this, together with the improvement of water quality, contributes to the intensive development of marine flora and fauna. As a result, an increase in the bioaccumulation of Hg in the trophic webs is observed (Bełdowska 2015; Bełdowska and Kobos 2016; Bełdowska et al. 2016).

Plant microorganisms in phytoplankton and phytobenthos are characterised by high metabolic rates and thus they rapidly assimilate chemical substances, including nutrients and toxic metals such as Hg. Although photosynthetic microorganisms forming biofilm are important first level in the trophic web, it is understudied in terms of mercury bioaccumulation, especially in the Baltic. Most of its biomass is comprised of diatoms (Witkowski 1993). Hence, the current study has been undertaken in order to estimate variation of Hg concentration in the microorganisms of various ecological formations including the epilithon, the epiphyton and plankton. The research not only takes into account the type of organism but also the function it performs. This problem is especially important in estuaries, because of the intense growth of marine organisms, some of which being commercial species, also for human consumption.

## Material and Methods

### Sample Collection

**Fig. 1:**
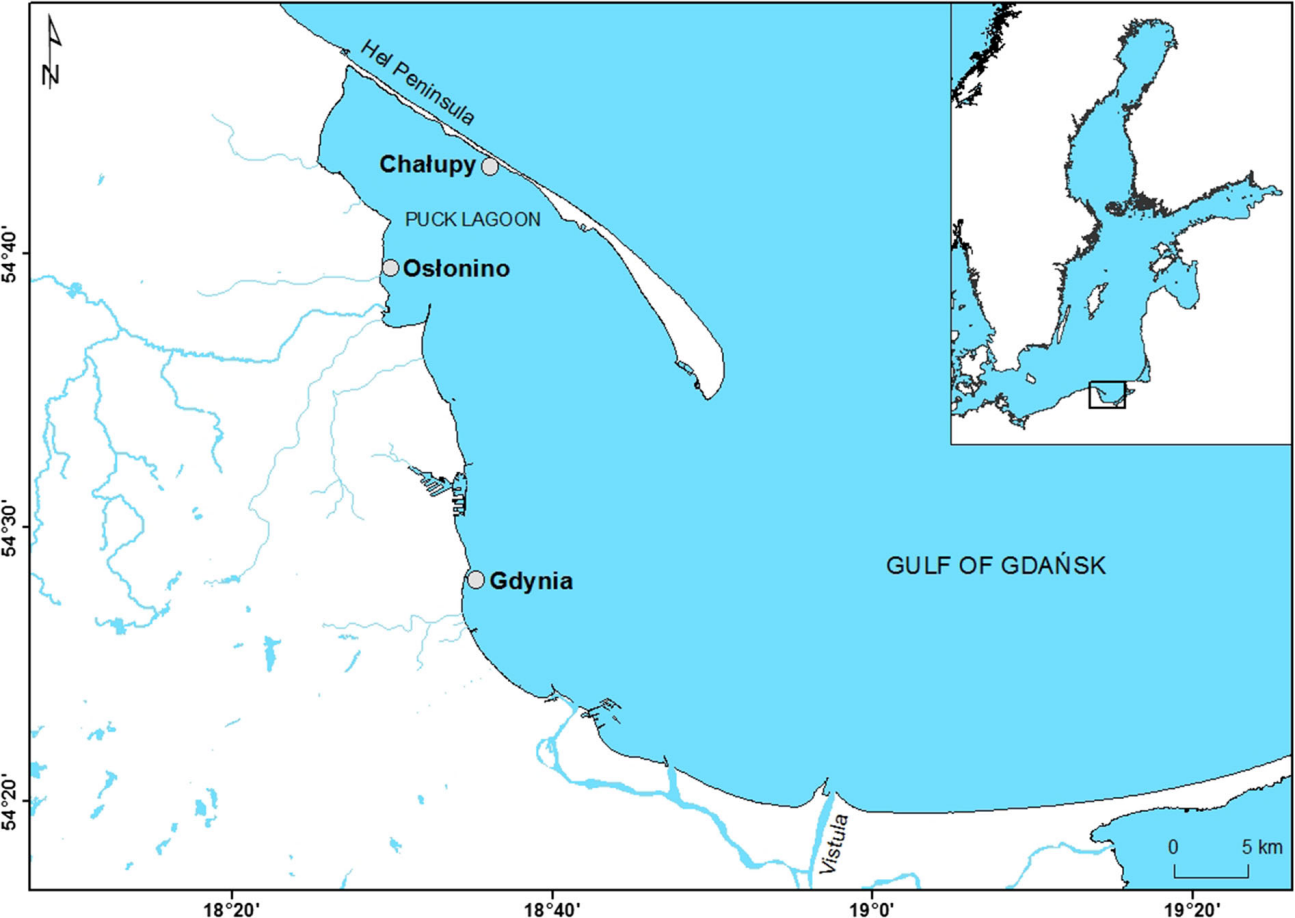
Map of sampling stations

Samples for analysis were collected once a month at three stations situated in the coastal zone of the Gulf of Gdańsk in the vicinities of Chałupy, Osłonino and Gdynia, between December 2011 and May 2013 (Gdynia to November 2012) (Fig. 1). Because of heavy icing, no samples were taken in February 2012, in Osłonino from January to March 2013 and in Chałupy from December 2012 to January 2013. Samples were collected from a depth of about 0.5 m. The research stations at Osłonino and Chałupy were located far from urban centres, in a region of little attraction to tourists. The station at Osłonino was in the immediate vicinity of the land (Fig. 1), coming under its influence due to the presence of numerous river estuaries (Reda, Gizdepka, Zagórska Struga), drainage channels and coastal erosion (Osłonino cliff). At the station at Chałupy, located on the opposite shore of the Puck Lagoon (Fig. 1) by the narrow strip of land known as the Hel Peninsula, the impact of the land was considerably less. The station at Gdynia, meanwhile, was located in close proximity to the urban and tourist-oriented Tricity agglomeration. Pollution from Gdynia was introduced into the sea by means of, among other channels, the River Kacza (which flows through Gdynia) and rainwater drainage channels. The coastal area at the Gdynia station was characterised by high environmental dynamics, especially compared to the other two stations located in the inner part of the Puck Lagoon (Nowacki 1993).

Material for the analysis of the epilithon was collected from the bottom of a permanent substrate (e.g. rocks) according to a methodology widely used in the monitoring of flowing waters (Kelly and Zgrundo 2013). Each stone was first gently rinsed with seawater and then strands of filamentous seaweed were peeled off as necessary. Finally, the biofilm from the surface of the stone was scraped off with a little brush. Samples of macrophytobenthos were collected using a manual Van Veen grab sampler with a grab area of 250 cm^2^ in three repeats. In order to separate the benthic organisms, samples of sediments were sifted through a net of 0.5-mm pore size. The epiphyton (biofilm from plants) was removed in the laboratory by ultrasound. The epiphyton and epilithon material collected for qualitative and quantitative biological analyses was placed in plastic containers, treated with Lugol’s solution or frozen for chemical analysis.

Additionally, every time, phytoplankton samples were collected. This was achieved using 20-μm nets. The samples for microscopic analyses of phytoplankton were preserved with Lugol’s solution (1%) and stored under cool and dark conditions, whereas for chemical measurements, they were frozen.

### Chemical Analyses

The collected samples were freeze-dried and analysed by means of the thermo-desorption advanced mercury analyser (AMA 254). The detection limit for solid materials was 0.005 ng g^−1^. QA/QC included blank samples, replicates and reference materials. All measurements of Hg in the reference materials were within the certified ranges (BCR 414 plankton and GBW 07314 sediment). Average errors did not exceed 5%. Details of the analysis were described by Bełdowska and Kobos (2016).

### Biological Analyses

The collected epilithon was placed in 150-ml beakers and rinsed two times with distilled water in order to remove Lugol’s solution and sea salt. Preparation of the diatoms was carried out using the modified method of Zgrundo et al. (2013). Around 10 ml of 69% H_2_SO_4_ was poured into each of the beakers and the contents were then boiled on a heating plate at about 115 °C to mineralise organic matter (for approx. 1 h). The material was then rinsed five times with distilled water. Afterwards, the diatom material was permanently fixed with Naphrax resin in glass slides.

Analysis of the diatoms in the fixed samples was performed using a Nikon 80i light microscope at × 1000 magnification using Nomarski contrast. In each sample, at least 350 diatoms were identified in terms of specie, genera or lowest possible taxonomic level and quantified. For diatoms, identification following taxonomic literature was used: Snoeijs 1993, Snoeijs and Vilbaste 1994, Snoeijs and Potapova 1995, Snoeijs and Kasperovičiene 1996, Snoeijs and Balashova 1998, Witkowski et al. 2000, Levkov 2009 and Hofmann et al. 2013. Microphotographic documentation was carried out simultaneously using a Nikon DS-U2 camera. Data from three samples (Gdynia 01/2012, 03/2012 and 04/2012) was not taken into account during statistical analysis due to insufficient coverage. Epiphyton was not qualitatively and quantitatively analysed due to methodological problems.

Qualitative analysis of phytoplankton was carried out using a Nikon Eclipse E200 light microscope at magnification of ×200, ×400 and ×630. The taxonomic literature included Derbes 1974, Pankov 1990 and Pliński and Witkowski 2009, 2011. For quantitative analysis, samples were left to settle for 24 h in 10- or 25-ml chambers depending on the amount of phytoplankton. Research was then performed using a Nikon TMS inverted microscope at magnification of ×100, ×200 and ×400 in accordance with the procedure set out by HELCOM PGE (HELCOM COMBINE 2014). Phytoplankton biomass was calculated according to the procedure described in Olenina et al. (2006) and Napiórkowska-Krzebietke and Kobos (2016).

### Processing Results

The bioconcentration factor (BCF) was calculated following the formula suggested by Szefer et al. (1999):

1 $$ \mathrm{BCF}={C}_{\mathrm{org}}/{C}_{\mathrm{water}} $$

where *C*_org_ (ng/kg dry weight (dw)) is the concentration of Hg in organisms from the different ecological formations (epilithon, epiphyton, phytoplankton) and *C*_water_ (ng/L) is the concentration of Hg in water. Mercury concentration in seawater was based on Bełdowska and Kobos (2016).

Information on life forms, biovolume and diatom ecological guilds was taken primarily from Rimet and Bouchez (2012) and Snoeijs (1993), Snoeijs and Vilbaste (1994), Snoeijs and Potapova (1995), Snoeijs and Kasperovičiene (1996) and Snoeijs and Balashova (1998). The guilds distinguished for diatoms in epilithon can be termed function groups. These are groups of organisms that inhabit the same habitat, but which adapted in different ways to the prevailing abiotic conditions. For the ‘low-profile’ guild in epilithon, Rimet and Bouchez (2012) counted diatoms of short stature including prostrate, adnate and erect diatoms which are resistant to physical disturbances (water turbulence) and do not tolerate nutrient enrichment. The ‘high-profile’ guild was established for large species or those which tend to form colonies. Diatoms from this group do not resist turbulence in the environment. The ‘motile’ guild consists of fast-moving species which adapted to the turbulent environment (Online Resource 1).

Statistical tests were performed using Statistica 10 computer software. The analysed data distribution was found to be non-parametric (Shapiro-Wilk test, *p* < 0.05). In order to determine the significance of differences between data, the Mann-Whitney *U* test was used. For all analyses, the level of significance *p* was ≤ 0.05.

## Results and Discussion

### Diatom Analysis

#### Diatoms in Epilithon

**Fig. 2:**
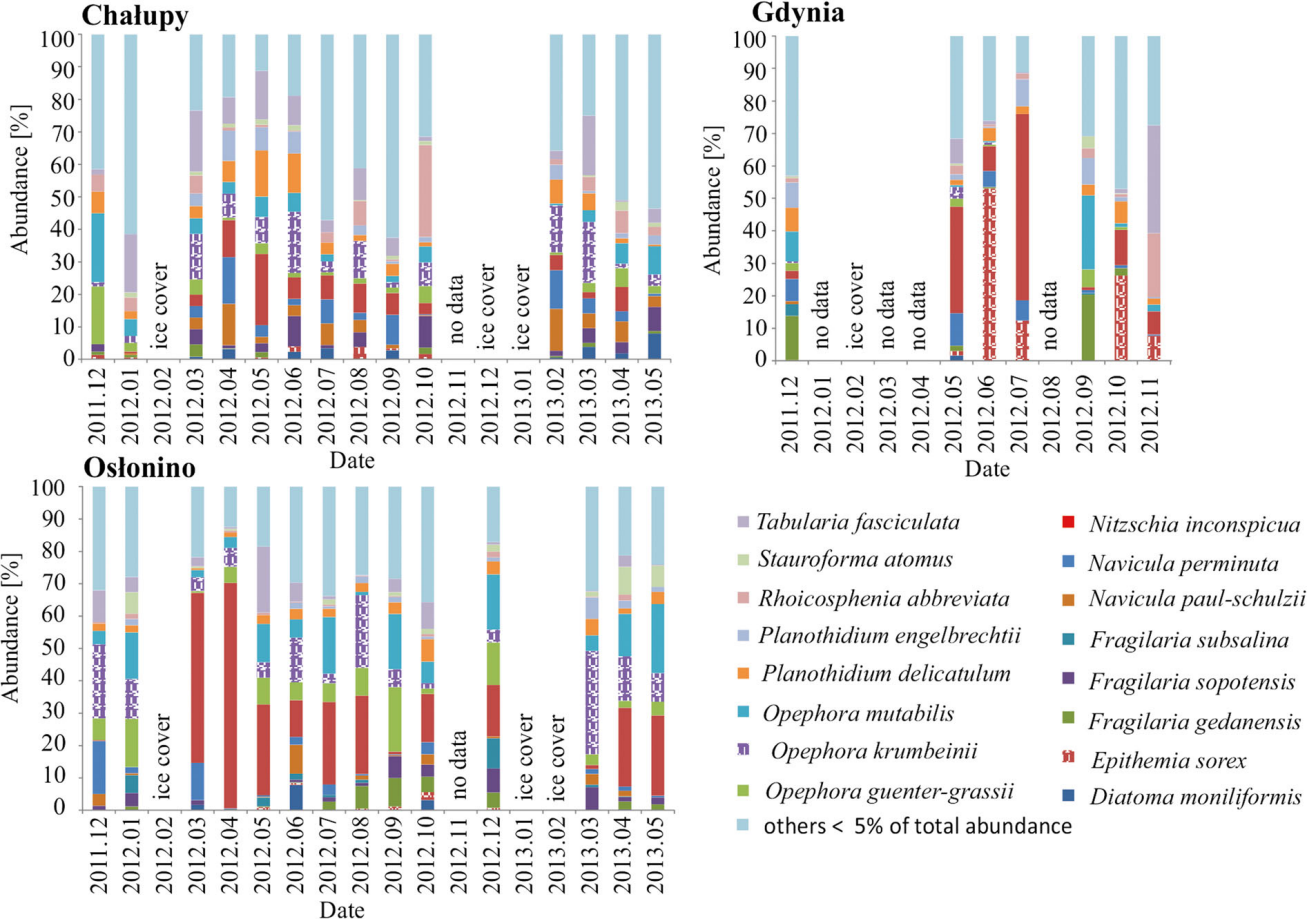
Share of dominant species of epilithic diatoms (at least 5% of total share in at least one sample) at stations in particular months

A total of 210 diatomic taxa (Online Resource 2) were identified in the analysed samples of epilithon from the Gulf of Gdańsk coastal zone. Most of the identified diatoms are constantly present in the waters of the gulf (Witkowski et al. 2000; Lange-Bertalot et al. 2003; Zgrundo et al. 2008; Majewska et al. 2012). All species were cosmopolitan in character and have been observed both in the Baltic and in the world oceans (Hällfors et al. 1981; Witkowski et al. 2000; Arrhenius et al. 2014; Guiry and Guiry 2017). Benthic taxa were found to be dominant (94% of all identified taxa), and 15 species occurred in more than half of the samples (Fig. 2, Online Resource 2). The most frequent were the following seven species: *Planothidium delicatulum* (Kützing) Round and Bukhtiyarova (97% of samples), *Opephora mutabilis* (Grunow) Sabbe and Vyverman and *Tabularia fasciculata* (Agardh) Williams et Round (94% of samples), *Opephora guenter-grassii* (Witkowski and Lange-Bertalot) Sabbe and Vyverman (92% of samples), *Navicula perminuta* Grunow in Van Heurck and *Nitzschia inconspicua* Grunow (89% of samples) and *Opephora krumbeinii* Witkowski, Witak and Stachura (86% of samples).

Planktonic diatoms accounted for 6% of the identified taxa in epilithon samples. Their small contribution (along with meroplankton) to epilithon communities suggests that this formation is a separate functional unit whose representatives do not change habitat, despite opportunities created by the environment, such as small depths, 0.5 m. Among those identified, 13 species were of the genera: *Aulacoseira*, *Chaetoceros*, *Conticribra*, *Cyclotella*, *Cyclostephanos*, *Skeletonema*, *Stephanodiscus* and *Thalassiosira* (Online Resource 3)*.* They occurred in small amounts—typically accounting for less than 0.5% of the total abundance. Hence, it can be concluded that planktonic diatoms were present in epilithon samples only by coincidence and had no influence on the functioning of their communities. An exception was noted in December 2011 at Osłonino, when *Cyclotella atomus* Hustedt formed a 6% share. However, this was a period of so-called withdrawal—an inflow of waters from deeper areas of the Gulf of Gdańsk—and this forced them landwards.

The stations were characterised by specific composition and structure of diatom communities in the epilithon. Communities in the Puck Lagoon (at the Chałupy and Osłonino stations) were more similar to each other, than to that of the Gdynia station (Fig. 2, Online Resource 2). There were 21 species identified, mainly high- and low-guild profiles (11 species), which were found at both Chałupy and Osłonino (Puck Lagoon), but not at the station in Gdynia. One of them, *Fragilaria sopotensis*, accounted for more than 5% of total abundance. In Gdynia, 35 of the identified species appeared only at this station—they were not present at either Osłonino or Chałupy. The majority of these (20 taxa) were of motile guild (Online Resource 2).

Seasonal variability was also observed at the individual stations. During the cold season, communities were comprised mainly of taxa preferring lower temperatures and more dynamic water mixing, such as *Opephora guenter-grassii* and *Planothidium engelbrechtii*.

#### Diatoms in Plankton

**Fig. 3:**
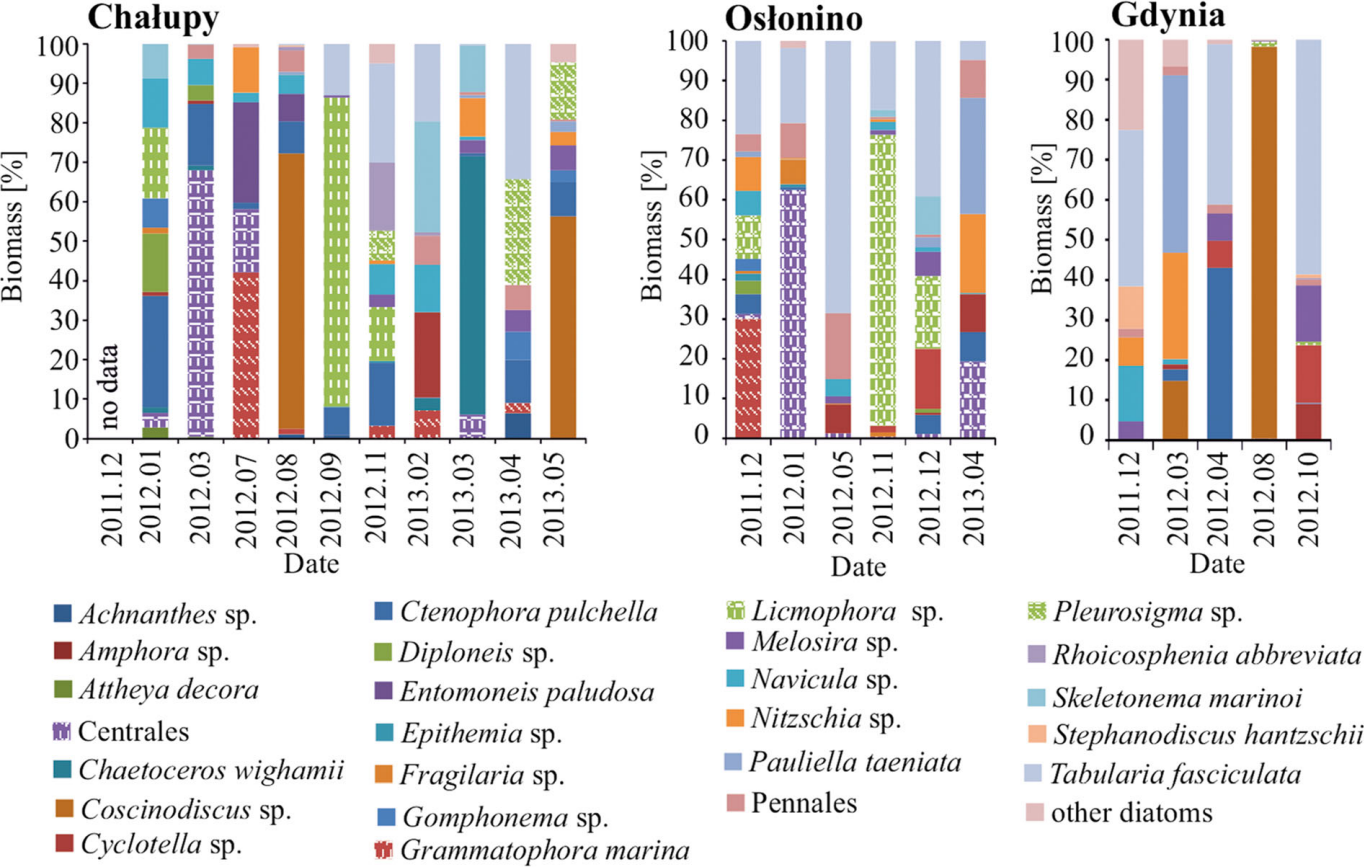
Share of diatoms in total biomass of phytoplankton at stations, in months when diatoms were predominant in the phytoplankton biomass

One hundred forty-three taxa were identified in plankton, of which 51 taxa were diatoms. In the spring and autumn, diatoms accounted for over 70% of the total biomass of phytoplankton (Fig. 3). A relatively large number of taxa (22) of higher benthic origin were observed among the diatoms present in phytoplankton communities (in Chałupy 1–10, average 7; in Osłonino 1–12, average 6; in Gdynia 3–9, average 5). The most frequently observed species of this group at all stations was *Tabularia fasciculata* (Agardh) Williams et Round. The group of epilithic diatoms observed periodically in plankton included *Diatoma tenuis* Agardh, *Melosira moniliformis* (O.F.Müller) C.Agardh and *Melosira nummuloides* C.Agardh. Their percentage abundance was considerably low: from 0 to 2%.

In terms of taxonomic richness, the stations at Chałupy and Osłonino were very similar. In Gdynia, both the maximum and the average number of taxa were slightly lower. The Gdynia station was also different from the Chałupy and Osłonino stations in terms of taxonomic composition—species which were not present in samples from the Puck Lagoon, such as *Licmophora* sp. and *Rhoicosphenia abbreviata*, occurred there frequently, while on the other hand, *Ulnaria ulna* was not observed.

Benthic diatoms bind to the substrate using extracellular polymeric substances (EPS). With their help, they slide along the underwater surfaces or attach to them, creating structures which resemble little cushions or various types of stalk (Round et al. 2007). The presence of benthic taxon in plankton is connected with the removal of their cells from the substrate as a result of mechanical factors, e.g. strong waves. It can therefore be assumed that the more benthic diatoms there are in plankton, the stronger the dynamics of the environment in which their communities develop.

### Hg in Epilithon

Median concentrations of Hg in the epilithon at the stations were similar and did not differ significantly (Mann-Whitney *U* test, *p* ≤ 0.05). The lowest median and mean concentration (24 ng g^−1^; 27 ng g^−1^) of mercury was in Chałupy, and the highest in Osłonino (31 ng g^−1^; 34 ng g^−1^) (Table 1). The bioconcentration factor of Hg from water (BCF) by epilithon and epiphyton was 10^3^ (Eq. 1). It was two, three times higher than BCF in macrophytobenthos form Gulf of Gdańsk (Bełdowska et al. 2015). The accumulation of Hg from water by the biofilm covering the stones and plants was confirmed by direct proportional correlations of mercury (Table 2). Epilithon from the station at Chałupy was an exception to this.

**Table 1:** Median (mean) of Hg concentration (ng g^−1^) in various ecological formations and in suspended particulate matter (SPM), sediment

	Chałupy	Osłonino	Gdynia	References
Epilithon	24 (27)	31 (34)	27 (29)	this study
Epiphyton	57 (58)	66 (62)	No data	this study
Phytoplankton	42	70	52	Bełdowska and Kobos 2016
Zooplankton	66	78	70	Bełdowska and Mudrak-Cegiołko 2017
Macroalgae	15	16	No data	Bełdowska et al. 2016
Angiosperms	8	8	No data	Bełdowska et al. 2016
SPM	55	48	60	Jędruch et al. 2017
Sediment	0.7	2.6	0.9	Bełdowski et al. 2018

**Table 2:** Statistically significant (*p* ≤ 0.05) linear correlation of Hg concentration in epilithon and other elements of the environment

	Chałupy	Osłonino	Gdynia
Seawater	–	0.7	0.5
Epiphyton	0.8	0.8	No data
Phytoplankton	–	–	–
Planktonic diatoms	–	0.97	–

Diatoms dominate in the biomass of both epilithon and epiphyton (Round et al. 2007; Arrhenius et al. 2014), hence the statistically significant correlation between the concentration of Hg in these formations at both stations in the Puck Lagoon (*r* = 0.8; *p* ≤ 0.05) (Table 2). There was no correlation between Hg concentration in epilithon and phytoplankton. However, during the period when the biomass of phytoplankton in the area of Osłonino was strongly dominated by diatoms, there was a correlation coefficient of *r* = 0.97 (*p* ≤ 0.05). This demonstrates a similar rate of Hg accumulation by planktonic diatoms and those from the epilithon.

**Fig. 4:**
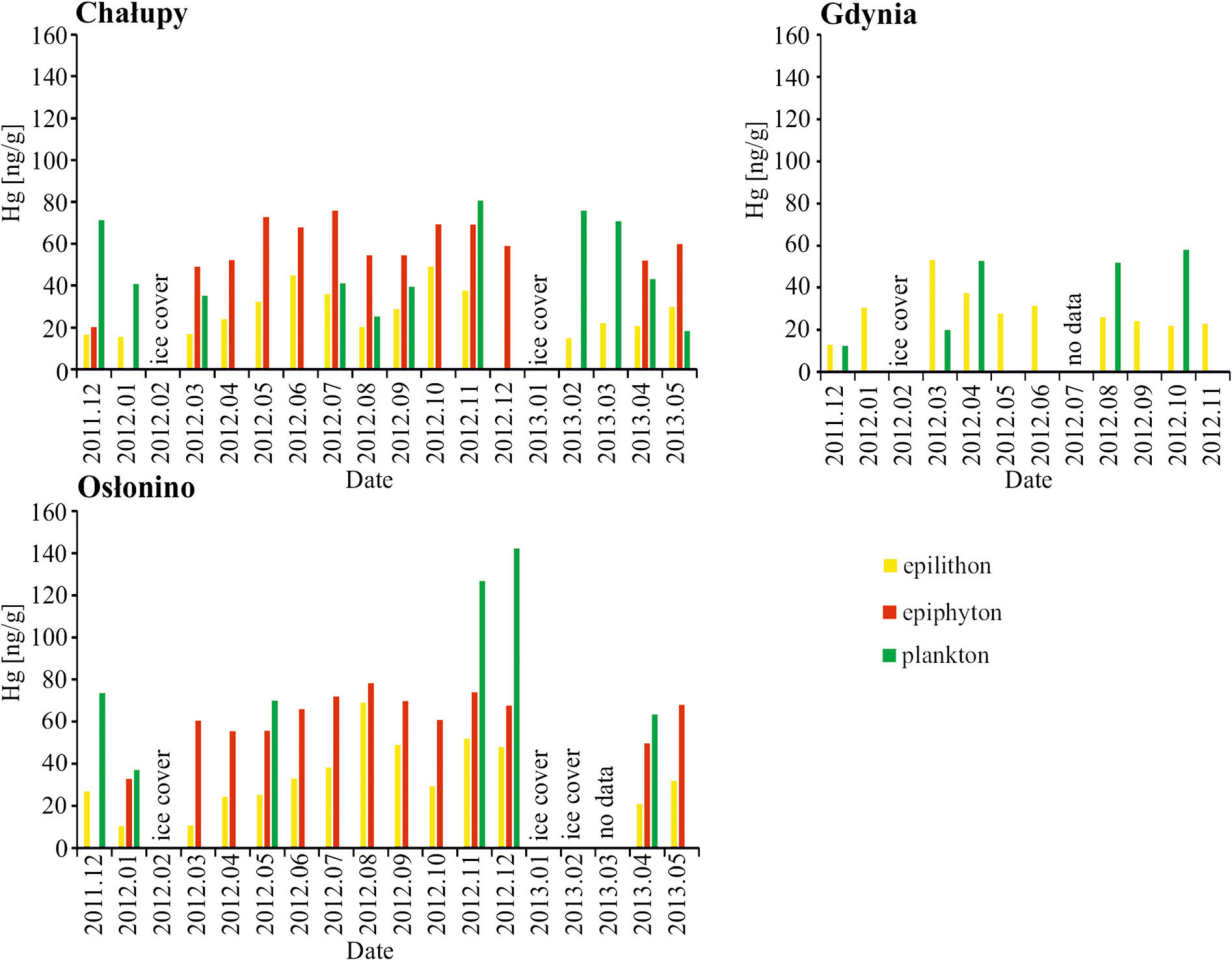
Hg concentration in epilithon, epiphyton and plankton during periods when diatoms were dominant in biomass

**Fig. 5:**
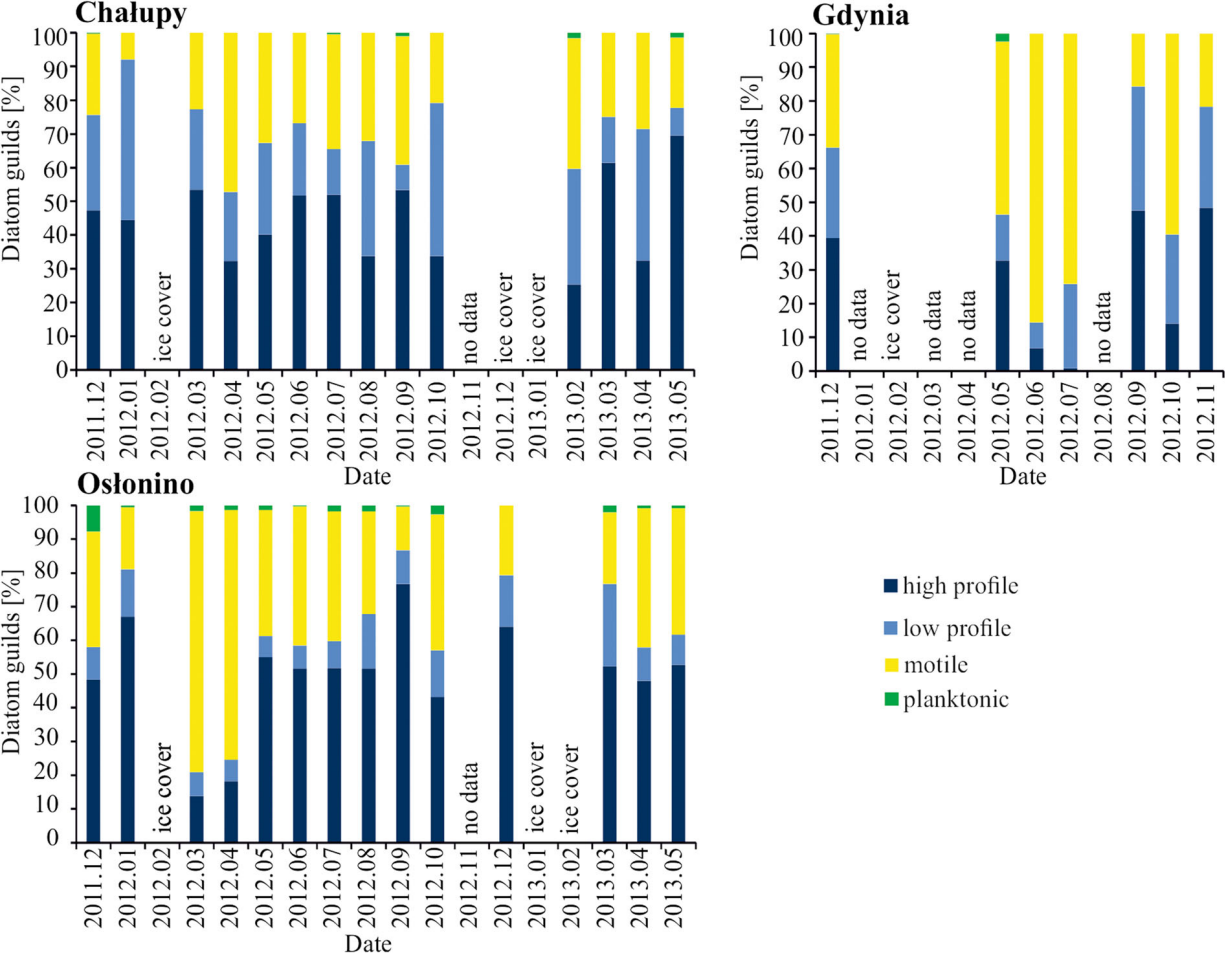
Percentage share of diatomic guilds in epilithon in particular months

Hg concentration in epilithon was two times lower than in plankton and epiphyton but up to 30 times higher than in sediments (Jędruch et al. 2015; Bełdowski et al. 2018) (Table 1; Fig. 4). Previous research indicates that Hg concentration in biofilm-covered macrophytes is influenced by water temperature and level, dissolved organic carbon and oxygen, available light, as well as host species (Hamelin et al. 2015a). In the studied area, the dominance of species belonging to the motile and high-profile guilds indicates a relatively high proportion of sediment in the biofilm formed by organisms in the epilithon (Fig. 5). Under conditions of relatively high sedimentation rate, organisms that form special structures high above the substrate or can actively move on the surface of accumulated sediment are more competitive in access to the key abiotic factor of light. In the open part of the gulf (Gdynia) where the waves were intensive, the dominant diatoms were of motile guild profile, i.e. those adapted to a turbulent environment (Rimet and Bouchez 2012). In the region of Chałupy, where the waves were less intensive but there was a clear influence of currents from the open sea, there were more or less equal numbers of high-profile and motile guild representatives. In Osłonino, in the most sheltered part of the Puck Lagoon, the low water dynamics and stable conditions favoured the steady development of the biofilm by dominant high-profile guild which, in winter, was swept away and replaced by motile.

#### Station Under the Influence of Bottom Currents—Chałupy

The highest concentrations of Hg in the epilithon of the Chałupy region were measured in June 2012 when *Opephora krumbeinii* (high profile) and *Planothidium delicatulum* were prevalent, and in October 2012 when *Rhoicosphenia abbreviata* (low profile) dominated (Figs. 2 and 5). In contrast, the maximum Hg concentrations in planktonic diatoms were recorded in November 2012 when the prevalent species were *Ctenophora pulchella*, *Licmophora* sp., *Rhoicosphenia abbreviata* and *Tabularia fasciculata* (all high-profile diatoms), in February 2013 when there were large shares of *Cyclotella choctawhatcheeana*, *Skeletonema marinoi* (both planktonic) and *Tabularia fasciculata*, in March 2013 when *Chaetoceros wighamii* (planktonic) was dominant, and in December 2011 when *Centrales (*planktonic), *Licmophora* sp. and *Navicula* sp. were prevalent (Figs. 3 and 5).

In the Chałupy region, an increase in mercury concentration in epilithon was observed during the warm months—from May to the freezing period (Fig. 4). During this period, the concentration of Hg increased with the decreasing percentage of motile guild organisms (*r* = − 0.8; *p* < 0.05) and the increasing share of high and low guild profiles (*r* = 0.8; *p* < 0.05). In August alone, the Hg concentration in diatoms (both in epilithon and epiphyton) was observed to drop by almost two times. It is likely that this phenomenon was directly caused by mass blooms of phytoplankton during the growing season. Dead flora contributed to the onset of anaerobic conditions (eh = − 435 eV (Bełdowski et al. 2018) and the appearance of hydrogen sulphide, and this change in abiotic conditions had a negative impact on organism development. The limited light exposure brought about by the excessive algae growth also negatively affected the development of biofilm. On the other hand, these were favourable conditions for mercury methylation. According to the previous reports, MeHg production could be even up to two times higher in periphyton than in local sediment (Hamelin et al. 2015b). Therefore, we can expect that despite the decreased of the total Hg concentration, there is a possibility that the contribution of MeHg increased.

Taking into account the entire study period, the Hg concentration in epilithon in the region of Chałupy demonstrated an inverse correlation with the share of the low-profile guild (*p* = − 0.7). This is probably due to the fact that this guild is the least competitive in terms of access to both light and soluble compounds in the water, and that such organisms are exposed to covering by marine sediments, thereby limiting their adsorption surface.

#### Station in an Area of Limited Water Exchange—Osłonino

The highest concentration of Hg in the epilithon was measured in August (Fig. 4). At that time, the concentration of Hg in the epiphyton was also at its highest. The dominant species were found to be (47%) *Opephora krumbeinii* (as in Chałupy, high profile) and *Nitzschia inconspicua* (motile profile) (Fig. 2). In contrast, the maximum Hg concentrations in planktonic diatoms were noted in November when *Licmophora* sp. (high profile) was clearly dominant (as was the case in Chałupy), and in December 2012 when two species also seen at Chałupy were prevalent: *Tabularia fasciculata* and *Licmophora* (both high profile) (Figs. 2 and 4).

In Osłonino, the concentration of Hg in the epilithon was analogous to that of Chałupy, being higher in the warmer months: it increased in spring and remained at elevated levels until late autumn (Fig. 4). This is a shallow region with limited water dynamics, sheltered on three sides by land (Fig. 1), and there is therefore a small spread of pollutants transported from the land. At this station, the concentration of Hg in the tested elements of the environment was greater in comparison with the station at Chałupy, where currents from the open sea have a significant influence, or in Gdynia, where there is intensive exchange of water with the open part of the bay (Table 1). Statistically significant (*p* < 0.05) correlations were observed between Hg concentration in epilithon and in epiphytic and planktonic diatoms (Table 2). Hg concentrations in the epilithon increased together with increased abundance of high-profile (*r* = 0.6) and low-profile guilds (*r* = 0.7) and a decrease in motile guild organisms (*r* = − 0.7).

#### Station in an Area of Strong Waves—Gdynia

In the area of Gdynia, epilithon samples were poor in diatoms. This was probably due to the strong water dynamics, which prevented the development of epilithon by constant mechanical removing it from the substrate. During the whole study period, the concentration of Hg in the epilithon was close to the median. The highest value was measured in March when *Pauliella taeniata* (planktonic) was clearly dominant and, as in Osłonino, *Nitzschia* sp. (Figs. 2 and 4). High Hg concentration was measured in phytoplankton diatoms in October, when similarly as in Chałupy and Oslonino *Tabularia fasciculata* (high profile) was clearly prevalent, in August when *Coscinodiscus granii* (planktonic) held a 93% share, and in April when (as in Chałupy) the predominant species were *Ctenophora pulchella* and *Tabularia fasciculata* (also the case in Osłonino, both high profile).

In this region, in contrast to the Puck Lagoon, the Hg concentration correlated directly with the motile guild (*r* = 0.5, *p* < 0.05) which predominated in diatoms (Fig. 5) and was inversely proportional to the low-profile guild (*r* = − 0.8, *p* < 0.05). This was most likely due to the effect of strong waves, which cause continuous tearing up and covering with sediment of organisms from the high- and low-profile guilds. Mobile microorganisms such as diatoms from the motile guild can remain in this dynamic zone for a long time and can easily re-colonise the habitat from which they were removed.

## Summary

Biofilm developing on the sea bottom and plants is an important level in the trophic web. It is epilithon and epiphyton, and not macroalgae, which comprise the main dietary components of herbivorous macrozoobenthos and fish, and so biofilm must therefore be regarded as an important vector in the transmission of nutrients, as well as toxic substances including mercury, to higher trophic levels. This is significant given that the concentration of Hg in epiphyton was four times higher than that of macroalgae and eight times that of angiosperms, while in the epilithon, it was twice as high as that of macroalgae and three times that of angiosperms (Saniewska et al. 2010; Bełdowska et al. 2016). The bioconcentration factor of the examined microorganisms was 10^3^ and it was two to three times higher than in the macrophytobenthos (Bełdowska et al. 2015). Methylmercury (the most toxic form of mercury) can constitute up to 74% of Hgtot in biofilm (Hamelin et al. 2015a). Diatoms, an essential component of phytoplankton, epilithon and epiphyton biomass, easily accumulate Hg from the environment, especially in shallow lagoons with low water dynamics. Biodiversity was observed to increase in the epilithon during the summer months and the concentration of Hg in biofilm increased in tandem with this. With phytoplankton, it was observed that together with an increase in biomass, there was biodilution of Hg (Bełdowska and Kobos 2016). However, as in the studies of Hamelin et al. (2015a), this process was not observed in epilithon or epiphyton. In this case, the increase in temperature was conducive to the accumulation of Hg in the first level of the trophic web. This is important in the southern Baltic region in light of climate warming, especially with regard to the cold season (late autumn–winter–early spring), and the lack of icing in the coastal zone in winter (HELCOM 2013). This extends the vegetation period for diatoms and therefore also the period of Hg accumulation, which in turn leads to an increase in the annual mercury load that is introduced into the trophic web.

The most effective accumulation of Hg in diatoms, under both low and high water dynamics, was observed when *Tabularia fasciculata* dominated. The concentration of mercury also increased in biofilm when *Ctenophora pulchella*, *Nitzschia* sp., *Licmophora* and *Opephora krumbeinii* were prevalent and when *Rhoicosphenia abbreviata* occurred in epilithon and phytoplankton. These are mainly organisms from the high-profile guild which, have a greater surface area through which to adsorb Hg. However, in a coastal area with intense waves where low- and high-profile guilds were removed, the process of Hg accumulation was instead observed in motile organisms.

Analysis of epiphytic diatoms was not performed as part of this study, but the species composition of epilithon and epiphyton is similar. In both cases, they comprise organisms adapted to live on a solid and stable substrate, hence the species composition, structure and functioning of such communities are practically identical (Snoeijs 1993; Snoeijs and Vilbaste 1994; Round et al. 2007). We can therefore assume that similar trends occur in the communities of microorganisms covering plants.

## Electronic Supplementary Material

ESM 1 (DOCX 68 kb)

ESM 2 (DOCX 71 kb)

ESM 3 (DOCX 22 kb)
