# Supplementary material for: Mercury in the Diatoms of Various Ecological Formations
Source: Water Air Soil Pollut. 2018 May 11;229(5):168. doi: 10.1007/s11270-018-3814-1 (PMC5945792; doi:10.1007/s11270-018-3814-1)

**Electronic Supplementary Material 1**

Mercury in the photosynthetic microorganisms, Archives of Environmental Contamination and Toxicology, Magdalena Bełdowska*, Aleksandra Zgrundo, Justyna Kobos; ^*^corresponding author: [m.beldowska@ug.edu.pl](mailto:m.beldowska@ug.edu.pl), Institute of Oceanography, University of Gdańsk


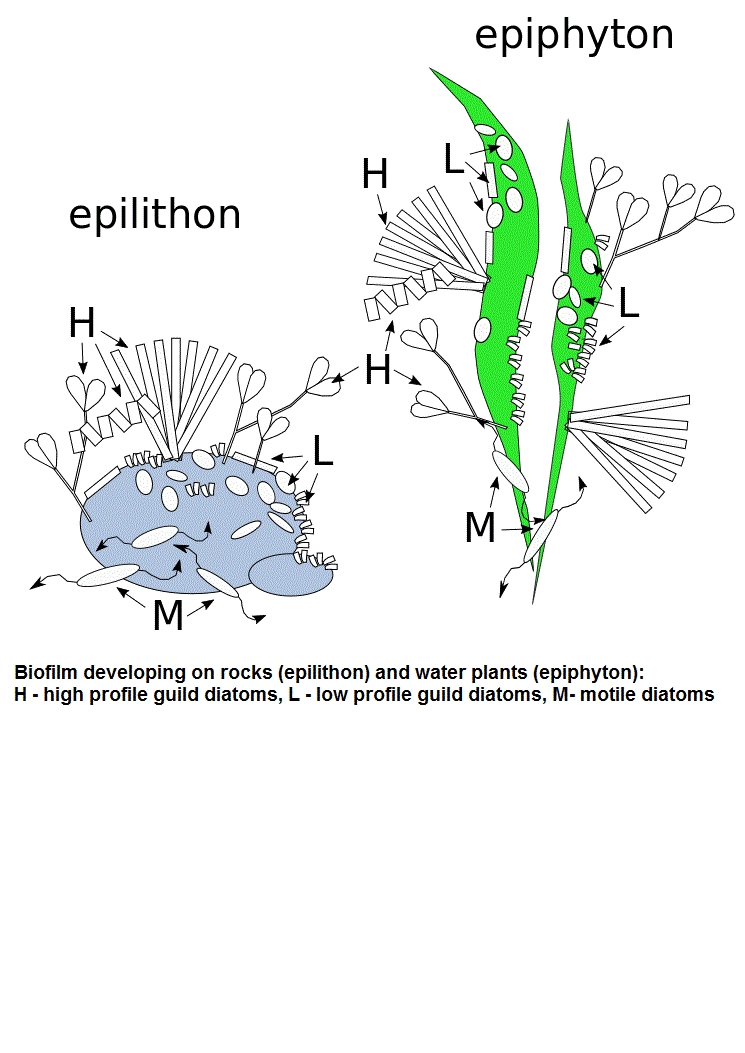

Supplement: Supplementary file 1 — (DOCX 68 kb) [file 11270_2018_3814_MOESM1_ESM.docx]
