# Supplementary material for: Mercury in the Diatoms of Various Ecological Formations
Source: Water Air Soil Pollut. 2018 May 11;229(5):168. doi: 10.1007/s11270-018-3814-1 (PMC5945792; doi:10.1007/s11270-018-3814-1)
Supplement: Supplementary file 2 — (DOCX 71 kb) [file 11270_2018_3814_MOESM2_ESM.docx]

**Electronic Supplementary Material 2**

Mercury in the photosynthetic microorganisms, Archives of Environmental Contamination and Toxicology, Magdalena Bełdowska*, Aleksandra Zgrundo, Justyna Kobos; ^*^corresponding author: [m.beldowska@ug.edu.pl](mailto:m.beldowska@ug.edu.pl), Institute of Oceanography, University of Gdańsk

Diatom taxa identified in epilithon samples

1 - present, 0 - absence

| **name + authorities** | **High profile guild** | **Low profile guild** | **Motile guild** | **Planktonic** | **Chałupy** | **Osłonino** | **Gdynia** |
| --- | --- | --- | --- | --- | --- | --- | --- |
| *Achnanthes brevipes* var. *brevipes* Agardh | 1 | 0 | 0 | 0 | 1 | 1 | 0 |
| *Achnanthes delicatissima* Simonsen | 0 | 1 | 0 | 0 | 1 | 1 | 0 |
| *Achnanthes minuscula* Hustedt | 0 | 1 | 0 | 0 | 1 | 0 | 1 |
| *Achnanthes* sp. J.B.M. Bory de St. Vincent | 0 | 1 | 0 | 0 | 0 | 0 | 1 |
| *Achnanthes spec.* C1 J.B.M. Bory de St. Vincent | 1 | 0 | 0 | 0 | 1 | 0 | 0 |
| *Achnanthes* spec. G1 J.B.M. Bory de St. Vincent | 0 | 1 | 0 | 0 | 0 | 0 | 1 |
| *Achnanthes* spec. O1 J.B.M. Bory de St. Vincent | 0 | 1 | 0 | 0 | 1 | 0 | 0 |
| *Achnanthes vistulana* Witkowski | 0 | 1 | 0 | 0 | 1 | 0 | 0 |
| *Achnanthidium minutissimum* (Kützing) Czarnecki | 0 | 1 | 0 | 0 | 1 | 0 | 0 |
| *Amicula specululum* (Witkowski) Witkowski | 0 | 0 | 1 | 0 | 1 | 1 | 1 |
| *Amphora* cf*. lineolata* Ehrenberg | 0 | 0 | 1 | 0 | 0 | 1 | 0 |
| *Amphora inariensis* Krammer | 0 | 1 | 0 | 0 | 1 | 1 | 1 |
| *Amphora ovalis* (Kützing) Kützing | 0 | 1 | 0 | 0 | 0 | 0 | 1 |
| *Amphora pediculus* (Kützing) Grunow | 0 | 1 | 0 | 0 | 1 | 1 | 1 |
| *Amphora* sp. Ehrenberg | 0 | 1 | 0 | 0 | 1 | 1 | 0 |
| *Amphora* spec. G1 Ehrenberg | 0 | 0 | 1 | 0 | 0 | 0 | 1 |
| *Amphora* spec. G2 Ehrenberg | 0 | 0 | 1 | 0 | 0 | 0 | 1 |
| *Astartiella bahusiensis* (Grunow) Witkowski, Lange-Bertalot & Metzeltin | 0 | 1 | 0 | 0 | 1 | 1 | 1 |
| *Astartiella bremeyeri* (Lange-Bertalot) Witkowski & Lange-Bertalot | 0 | 1 | 0 | 0 | 0 | 1 | 0 |
| *Astartiella punctifera* (Hustedt) Witkowski & Lange-Bertalot | 0 | 1 | 0 | 0 | 0 | 0 | 1 |
| *Astartiella* sp. Witkowski, Lange-Bertalot & Metzeltin | 0 | 1 | 0 | 0 | 1 | 1 | 0 |
| *Aulacoseira* sp. Thwaites | 0 | 0 | 0 | 1 | 0 | 1 | 0 |
| *Aulacoseira subarctica* (O.Muller) Haworth | 0 | 0 | 0 | 1 | 0 | 1 | 0 |
| *Bacillaria paxillifera* (O.F. Muller) Hendey var.paxillifera | 0 | 0 | 1 | 0 | 1 | 1 | 0 |
| *Berkeleya rutilans* (Trentepohl) Grunow | 1 | 0 | 0 | 0 | 1 | 1 | 1 |
| *Biremis lucens* (Hustedt) Sabbe, Witkowski & Vyverman | 0 | 1 | 0 | 0 | 0 | 0 | 1 |
| *Brebissonia lanceolata* (C.Agardh) Mahoney & Reimer | 1 | 0 | 0 | 0 | 0 | 1 | 0 |
| *Caloneis amphisbaena* (Bory) Cleve fo.amphisbaena | 0 | 0 | 1 | 0 | 1 | 0 | 0 |
| *Caloneis crassa* (Gregory) R.Ross | 0 | 0 | 1 | 0 | 0 | 0 | 1 |
| *Catenula adhaerens* (Mereschkowsky) Mereschkowsky | 0 | 1 | 0 | 0 | 1 | 1 | 1 |
| *Chaetoceros cf. fallax* Prosckina-Lavrenko | 0 | 0 | 0 | 1 | 0 | 0 | 1 |
| *Cocconeis hauniensis* Witkowski | 0 | 1 | 0 | 0 | 1 | 1 | 1 |
| *Cocconeis hoffmannii* Simonsen | 0 | 1 | 0 | 0 | 0 | 0 | 1 |
| *Cocconeis irregularis* (P.Schulz) Witkowski | 0 | 1 | 0 | 0 | 0 | 0 | 1 |
| *Cocconeis molesta* Kützing var. *molesta* | 0 | 1 | 0 | 0 | 0 | 1 | 1 |
| *Cocconeis neothumensis* Krammer | 0 | 1 | 0 | 0 | 1 | 1 | 1 |
| *Cocconeis pediculus* Ehrenberg | 0 | 1 | 0 | 0 | 1 | 1 | 1 |
| *Cocconeis peltoides* Hustedt | 0 | 1 | 0 | 0 | 1 | 1 | 1 |
| *Cocconeis placentula* Ehrenberg var. *placentula* | 0 | 1 | 0 | 0 | 1 | 1 | 1 |
| *Cocconeis scutellum* Ehrenberg | 0 | 1 | 0 | 0 | 1 | 1 | 1 |
| *Cocconeis* sp. Ehrenberg | 0 | 1 | 0 | 0 | 0 | 0 | 1 |
| *Conticribra weissflogii* (Grunow) Stachura-Suchoples & D.M.Williams | 0 | 0 | 0 | 1 | 0 | 1 | 0 |
| *Ctenophora pulchella* (Ralfs ex Kützing) Williams & Round | 1 | 0 | 0 | 0 | 1 | 1 | 0 |
| *Cyclostephanos dubius* (Fricke) Round | 0 | 0 | 0 | 1 | 0 | 1 | 0 |
| *Cyclotella atomus* Hustedt | 0 | 0 | 0 | 1 | 0 | 1 | 0 |
| *Cyclotella choctawhatcheeana* Prasad | 0 | 0 | 0 | 1 | 1 | 0 | 1 |
| *Cyclotella meneghiniana* Kützing | 0 | 0 | 0 | 1 | 0 | 1 | 0 |
| *Cymbella excisa* Kützing var. *excisa* | 0 | 1 | 0 | 0 | 1 | 0 | 0 |
| *Cymbella* sp. Agardh | 1 | 0 | 0 | 0 | 0 | 0 | 1 |
| *Denticula subtilis* Grunow | 0 | 0 | 1 | 0 | 0 | 0 | 1 |
| *Diatoma moniliformis* Kützing | 1 | 0 | 0 | 0 | 1 | 1 | 1 |
| *Diatoma tenuis* Agardh | 0 | 0 | 0 | 1 | 1 | 1 | 0 |
| *Dickieia subinflata* (Grunow) D.G.Mann | 0 | 0 | 0 | 0 | 1 | 0 | 0 |
| *Diploneis didyma* (Ehrenberg) Ehrenberg | 0 | 0 | 1 | 0 | 1 | 1 | 0 |
| *Diploneis smithii* (Brébisson) Cleve | 0 | 0 | 1 | 0 | 1 | 0 | 0 |
| *Diploneis stroemii* Hustedt | 0 | 0 | 1 | 0 | 0 | 1 | 0 |
| *Diploneis vacillans* (A.Schmidt) Cleve | 0 | 0 | 1 | 0 | 1 | 0 | 0 |
| *Eolimna minima* (Grunow) Lange-Bertalot | 0 | 0 | 1 | 0 | 0 | 1 | 0 |
| *Epithemia adnata* (Kutzing) Brebisson | 0 | 0 | 1 | 0 | 1 | 1 | 0 |
| *Epithemia sorex* Kützing | 0 | 0 | 1 | 0 | 1 | 1 | 1 |
| *Epithemia* sp. Kützing | 0 | 0 | 1 | 0 | 0 | 0 | 1 |
| *Epithemia turgida* (Ehrenberg) Kützing var. *turgida* | 0 | 0 | 1 | 0 | 0 | 0 | 1 |
| *Fallacia cassubiae* Witkowski | 0 | 0 | 1 | 0 | 0 | 1 | 0 |
| *Fallacia clepsidroides* Witkowski | 0 | 0 | 1 | 0 | 1 | 1 | 1 |
| *Fallacia cryptolyra* (Brockmann) Stickle & D.G.Mann | 0 | 0 | 1 | 0 | 1 | 1 | 1 |
| *Fallacia floriniae* (M.Møller) Witkowski | 0 | 0 | 1 | 0 | 1 | 0 | 1 |
| *Fallacia forcipata* (Greville) Stickle & D.G.Mann | 0 | 0 | 1 | 0 | 0 | 1 | 1 |
| *Fallacia* sp. Stickle et Mann | 0 | 0 | 1 | 0 | 0 | 0 | 1 |
| *Fallacia tenera* (Hustedt) D.G.Mann | 0 | 0 | 1 | 0 | 0 | 1 | 1 |
| *Fistulifera saprophila* (Lange-Bertalot & Bonik) Lange-Bertalot | 0 | 0 | 1 | 0 | 0 | 0 | 1 |
| *Fragilaria amicorum* Witkowski & Lange-Bertalot | 1 | 0 | 0 | 0 | 1 | 1 | 1 |
| *Fragilaria cassubica* Witkowski & Lange-Bertalot | 1 | 0 | 0 | 0 | 0 | 1 | 1 |
| *Fragilaria* cf. *capensis* Grunow | 1 | 0 | 0 | 0 | 0 | 0 | 1 |
| *Fragilaria eichornii* Witkowski & Lange-Bertalot | 1 | 0 | 0 | 0 | 1 | 0 | 0 |
| *Fragilaria gedanensis* Witkowski | 1 | 0 | 0 | 0 | 1 | 1 | 1 |
| *Fragilaria hyalina* (Kützing) Grunow | 1 | 0 | 0 | 0 | 1 | 1 | 0 |
| *Fragilaria improbula* Witkowski & Lange-Bertalot | 1 | 0 | 0 | 0 | 1 | 1 | 0 |
| *Fragilaria labei* Witkowski & Metzeltin | 1 | 0 | 0 | 0 | 1 | 0 | 0 |
| *Fragilaria perminuta* (Grunow) Lange-Bertalot | 1 | 0 | 0 | 0 | 1 | 0 | 0 |
| *Fragilaria sopotensis* Witkowski & Lange-Bertalot | 1 | 0 | 0 | 0 | 1 | 1 | 0 |
| *Fragilaria* sp. Lyngbye | 1 | 0 | 0 | 0 | 1 | 0 | 1 |
| *Fragilariforma virescens* var. *subsalina* (Grunow) L.N.Bukhtiyarova | 1 | 0 | 0 | 0 | 0 | 1 | 1 |
| *Gomphoneis exigua* (Kützing) Medlin | 1 | 0 | 0 | 0 | 1 | 0 | 0 |
| *Gomphonema olivaceum* var. *balticum* (Cleve) Grunow | 1 | 0 | 0 | 0 | 1 | 1 | 1 |
| *Gomphonema parvulum* (Kützing) Kützing var. *parvulum* f. *parvulum* | 1 | 0 | 0 | 0 | 1 | 0 | 0 |
| *Gomphonema* sp. Ehrenberg | 1 | 0 | 0 | 0 | 0 | 0 | 0 |
| *Grammatophora marina* (Lyngbye) Kützing | 1 | 0 | 0 | 0 | 0 | 1 | 1 |
| *Grammatophora oceanica* Ehrenberg | 1 | 0 | 0 | 0 | 0 | 0 | 1 |
| *Halamphora acutiuscula* (Kützing) Levkov | 0 | 0 | 1 | 0 | 0 | 0 | 1 |
| *Halamphora coffeiformis* (C.Agardh) Levkov | 0 | 0 | 1 | 0 | 1 | 1 | 1 |
| *Halamphora exigua* (W.Gregory) Levkov 2009 | 0 | 0 | 1 | 0 | 1 | 1 | 1 |
| *Halamphora luciae* (Cholnoky) Levkov | 0 | 0 | 1 | 0 | 0 | 0 | 1 |
| *Halamphora tenerrima* (Aleem & Hustedt) Levkov | 0 | 0 | 1 | 0 | 1 | 1 | 1 |
| *Haslea spicula* (Hickie) Bukhtiyarova | 0 | 0 | 1 | 0 | 1 | 0 | 0 |
| *Hippodonta arctica* (Patrick& Freese)Lange-Bertalot, Metzeltin & Witkowski | 0 | 0 | 1 | 0 | 0 | 0 | 1 |
| *Hippodonta hungarica* (Grunow) Lange-Bertalot Metzeltin & Witkowski | 0 | 0 | 1 | 0 | 0 | 0 | 1 |
| *Hippodonta* sp. Lange-Bertalot, Metzeltin & Witkowski | 0 | 0 | 1 | 0 | 0 | 1 | 1 |
| *Hyalosira parietina* Witkowski | 1 | 0 | 0 | 0 | 1 | 0 | 1 |
| *Hyalosira* sp. Kützing | 1 | 0 | 0 | 0 | 0 | 1 | 0 |
| *Karayevia clevei* (Grunow) Bukhtiyarova | 0 | 1 | 0 | 0 | 1 | 1 | 0 |
| *Karayevia submarina* (Hustedt) Bukhtiyarova | 0 | 1 | 0 | 0 | 1 | 0 | 0 |
| *Licmophora abbreviata* C.Agardh | 1 | 0 | 0 | 0 | 1 | 1 | 1 |
| *Licmophora* C.Agardh | 1 | 0 | 0 | 0 | 1 | 1 | 0 |
| *Licmophora dalmatica* (Kützing) Grunow | 1 | 0 | 0 | 0 | 1 | 0 | 0 |
| *Licmophora debilis* (Kützing) Grunow | 1 | 0 | 0 | 0 | 0 | 0 | 1 |
| *Luticola mutica* (Kützing) D.G. Mann | 0 | 0 | 1 | 0 | 1 | 0 | 0 |
| *Martyana schulzii* (C.Brockmann) Snoeijs 1991 | 1 | 0 | 0 | 0 | 1 | 1 | 1 |
| *Mastogloia baltica* Grunow | 0 | 0 | 1 | 0 | 1 | 0 | 0 |
| *Mastogloia exigua* F.W.Lewis | 0 | 0 | 1 | 0 | 1 | 0 | 0 |
| *Mastogloia pumila* (Grunow) Cleve | 0 | 0 | 1 | 0 | 1 | 1 | 0 |
| *Mastogloia pusilla* Grunow | 0 | 0 | 1 | 0 | 1 | 0 | 0 |
| *Mastogloia smithii* Thwaites ex W.Smith | 0 | 0 | 1 | 0 | 1 | 1 | 0 |
| *Mastogloia* sp. Thwaites ex W.Smith | 0 | 0 | 1 | 0 | 1 | 0 | 0 |
| *Melosira moniliformis* (O.F.Müller) C.Agardh | 1 | 0 | 0 | 1 | 0 | 1 | 1 |
| *Melosira nummuloides* C.Agardh | 1 | 0 | 0 | 1 | 1 | 1 | 1 |
| *Navicula aleksandrae* Lange-Bertalot, Bogaczewicz-Adamczak & Witkowski | 0 | 0 | 1 | 0 | 1 | 1 | 1 |
| *Navicula antonii* Lange-Bertalot | 0 | 0 | 1 | 0 | 0 | 0 | 1 |
| *Navicula bipustulata* D.G.Mann | 0 | 0 | 1 | 0 | 1 | 0 | 1 |
| *Navicula bozenae* Lange-Bertalot, Witkowski & Zgrundo | 0 | 0 | 1 | 0 | 1 | 0 | 1 |
| *Navicula cari* Ehrenberg | 0 | 0 | 1 | 0 | 0 | 0 | 1 |
| *Navicula* cf. *digitoconvergens* Lange-Bertalot | 0 | 0 | 1 | 0 | 1 | 0 | 0 |
| *Navicula* cf. *normaloides* Cholnoky | 0 | 0 | 1 | 0 | 0 | 0 | 1 |
| *Navicula* cf. *radiosafallax* Lange-Bertalot | 0 | 0 | 1 | 0 | 1 | 0 | 0 |
| *Navicula cincta* (Ehrenberg) Ralfs in Pritchard | 0 | 0 | 1 | 0 | 0 | 1 | 0 |
| *Navicula flanatica* Grunow | 0 | 0 | 1 | 0 | 1 | 1 | 1 |
| *Navicula germanopolonica* Witkowski & Lange-Bertalot | 0 | 0 | 1 | 0 | 1 | 1 | 1 |
| *Navicula gregaria* Donkin | 0 | 0 | 1 | 0 | 1 | 1 | 1 |
| *Navicula hanseatica* Lange-Bertalot & Stachura | 0 | 0 | 1 | 0 | 1 | 1 | 0 |
| *Navicula lanceolata* (Agardh) Ehrenberg | 0 | 0 | 1 | 0 | 0 | 1 | 1 |
| *Navicula meniscus* Schumann | 0 | 0 | 1 | 0 | 1 | 0 | 0 |
| *Navicula normaloides* Cholnoky | 0 | 0 | 1 | 0 | 0 | 1 | 1 |
| *Navicula palpebralis* Brébisson ex W.Smith | 0 | 0 | 1 | 0 | 0 | 1 | 0 |
| *Navicula paul-schulzii* Witkowski & Lange-Bertalot | 0 | 0 | 1 | 0 | 1 | 1 | 1 |
| *Navicula perminuta* Grunow in Van Heurck | 0 | 0 | 1 | 0 | 1 | 1 | 1 |
| *Navicula phyllepta* Kutzing | 0 | 0 | 1 | 0 | 1 | 0 | 0 |
| *Navicula pseudosalinarioides* Giffen | 0 | 0 | 1 | 0 | 0 | 1 | 0 |
| *Navicula radiosa* Kützing | 0 | 0 | 1 | 0 | 0 | 1 | 0 |
| *Navicula ramosissima* (C.Agardh) Cleve | 0 | 0 | 1 | 0 | 1 | 1 | 1 |
| *Navicula rhynchotella* Lange-Bertalot | 0 | 0 | 1 | 0 | 0 | 1 | 0 |
| *Navicula salinarum* Grunow in Cleve et Grunow var.salinarum | 0 | 0 | 1 | 0 | 1 | 0 | 0 |
| *Navicula salinicola* Hustedt | 0 | 0 | 1 | 0 | 1 | 1 | 1 |
| *Navicula* sp. 1 Bory | 0 | 0 | 1 | 0 | 1 | 1 | 1 |
| *Navicula* spp. Bory | 0 | 0 | 1 | 0 | 0 | 0 | 1 |
| *Navicula supergregaria* Rumrich & Lange-Bertalot | 0 | 0 | 1 | 0 | 1 | 1 | 1 |
| *Navicula tripunctata* (O.F.Muller) Bory | 0 | 0 | 1 | 0 | 0 | 0 | 1 |
| *Navicula veneta* Kützing | 0 | 0 | 1 | 0 | 1 | 0 | 0 |
| *Navicula viminoides* spp. *cosmomarina* Lange-Bertalot, Witkowski, Bogaczewicz-Adamczak, Zgrundo | 0 | 0 | 1 | 0 | 0 | 0 | 1 |
| *Nitzschia* aff. *hybrida* Grunow | 0 | 0 | 1 | 0 | 0 | 1 | 1 |
| *Nitzschia agnita* Hustedt | 0 | 0 | 1 | 0 | 1 | 0 | 1 |
| *Nitzschia aurariae* Cholnoky | 0 | 0 | 1 | 0 | 1 | 1 | 1 |
| *Nitzschia capitellata* Hustedt in A.Schmidt & al. | 0 | 0 | 1 | 0 | 1 | 1 | 0 |
| *Nitzschia dissipata* (Kützing) Grunow var. *dissipata* | 0 | 0 | 1 | 0 | 0 | 1 | 0 |
| *Nitzschia frequens* Hustedt | 0 | 0 | 1 | 0 | 1 | 0 | 0 |
| *Nitzschia frustulum* (Kützing) Grunow var. *frustulum* | 0 | 0 | 1 | 0 | 1 | 0 | 0 |
| *Nitzschia hungarica* Grunow | 0 | 0 | 1 | 0 | 0 | 0 | 1 |
| *Nitzschia inconspicua* Grunow | 0 | 0 | 1 | 0 | 1 | 1 | 1 |
| *Nitzschia liebetruthii* Rabenhorst var. *liebetruthii* | 0 | 0 | 1 | 0 | 0 | 1 | 0 |
| *Nitzschia linearis* (Agardh) W.M.Smith var. *subtilis* (Grunow) Hustedt | 0 | 0 | 1 | 0 | 0 | 1 | 0 |
| *Nitzschia microcephala* Grunow in Cleve & Möller | 0 | 0 | 1 | 0 | 1 | 1 | 1 |
| *Nitzschia palea* (Kützing) W.Smith | 0 | 0 | 1 | 0 | 0 | 1 | 0 |
| *Nitzschia perspicua* Cholnoky | 0 | 0 | 1 | 0 | 0 | 1 | 0 |
| *Nitzschia recta* Hantzsch in Rabenhorst | 0 | 0 | 1 | 0 | 0 | 1 | 0 |
| *Nitzschia* sp. Hassall | 0 | 0 | 1 | 0 | 1 | 1 | 1 |
| *Nitzschia thermaloides* Hustedt | 0 | 0 | 1 | 0 | 0 | 1 | 0 |
| *Nitzschia thermaloides* Hustedt | 0 | 0 | 1 | 0 | 0 | 0 | 1 |
| *Nitzschia tubicola* Grunow | 0 | 0 | 1 | 0 | 0 | 1 | 0 |
| *Nitzschia valdestriata* Aleem & Hustedt | 0 | 0 | 1 | 0 | 1 | 0 | 0 |
| *Opephora guenter-grassii* (Witkowski & Lange-Bertalot) Sabbe & Vyverman | 1 | 0 | 0 | 0 | 1 | 1 | 1 |
| *Opephora krumbeinii* Witkowski, Witak & Stachura | 1 | 0 | 0 | 0 | 1 | 1 | 1 |
| *Opephora mutabilis* (Grunow) Sabbe & Vyverman | 1 | 0 | 0 | 0 | 1 | 1 | 1 |
| Pennales | 0 | 0 | 1 | 0 | 1 | 1 | 0 |
| Pennales C1 | 0 | 0 | 1 | 0 | 1 | 0 | 0 |
| *Petroneis humerosa* (Brébisson ex W.Smith) Stickle & D.G.Mann | 0 | 0 | 1 | 0 | 1 | 0 | 0 |
| *Pinnularia* sp. Ehrenberg | 0 | 0 | 1 | 0 | 1 | 0 | 0 |
| *Placoneis clementis* (Grunow) Cox | 0 | 0 | 1 | 0 | 1 | 1 | 1 |
| *Placoneis protracta* (Grunow) Mereschkowsky | 0 | 0 | 1 | 0 | 0 | 1 | 0 |
| *Plagiotropis tayrecta* Paddock | 0 | 0 | 1 | 0 | 0 | 1 | 0 |
| *Planothidium delicatulum* (Kützing) Round & Bukhtiyarova | 0 | 1 | 0 | 0 | 1 | 1 | 1 |
| *Planothidium dispar* (Cleve) Witkowski, Lange-Bertalot & Metzeltin | 0 | 1 | 0 | 0 | 0 | 1 | 0 |
| *Planothidium engelbrechtii* (Cholnocky) Round & Bukhtiyarova | 0 | 1 | 0 | 0 | 1 | 1 | 1 |
| *Planothidium hauckianum* (Grun.) Round & Bukhtiyarova | 0 | 1 | 0 | 0 | 1 | 1 | 1 |
| *Planothidium lemmermannii* (Hustedt) Morales | 0 | 1 | 0 | 0 | 1 | 1 | 1 |
| *Planothidium rostratum* (Oestrup) Lange-Bertalot | 0 | 1 | 0 | 0 | 0 | 1 | 0 |
| *Pleurosigma salinarum* (Grunow) Grunow | 0 | 0 | 1 | 0 | 0 | 1 | 0 |
| *Pravifusus hyalinus* Witkowski | 1 | 0 | 0 | 0 | 1 | 0 | 0 |
| *Proschkinia bulnheimii* (Grunow) Karayeva | 0 | 0 | 1 | 0 | 0 | 1 | 0 |
| *Proschkinia complanata* (Grunow) D.G.Mann | 0 | 0 | 1 | 0 | 1 | 0 | 0 |
| *Psammothidium punctulatum* (Simonsen) Bukhtiyarova & Round | 0 | 1 | 0 | 0 | 0 | 1 | 1 |
| *Pseudostaurosira brevistriata* (Grunow in Van Heurck) Williams & Round | 1 | 0 | 0 | 0 | 0 | 1 | 0 |
| *Pseudostaurosira zeilleri* (Héribaud-Joseph) D.M.Williams & Round | 1 | 0 | 0 | 0 | 1 | 1 | 0 |
| *Pseudostaurosiropsis geocollegarum* (Witkowski) E.A.Morales | 1 | 0 | 0 | 0 | 1 | 0 | 1 |
| *Rhoicosphenia abbreviata* (C.Agardh) Lange-Bertalot | 0 | 1 | 0 | 0 | 1 | 1 | 1 |
| *Rhopalodia gibba* (Ehrenberg) O.Muller var. gibba | 0 | 0 | 1 | 0 | 1 | 1 | 1 |
| *Skeletonema marinoi* Sarno & Zingone | 0 | 0 | 0 | 1 | 1 | 1 | 0 |
| *Stauroforma atomus* (Hustedt) D.Talgatti, C.E.Wetzel, E.Morales & L.C.Torgan | 1 | 0 | 0 | 0 | 1 | 1 | 1 |
| *Staurosira construens* Ehrenberg | 1 | 0 | 0 | 0 | 0 | 0 | 1 |
| *Staurosira venter* (Ehrenberg) Cleve & Moeller | 1 | 0 | 0 | 0 | 1 | 1 | 1 |
| *Staurosirella lapponica* (Grunow) D.M.Williams & Round | 1 | 0 | 0 | 0 | 0 | 1 | 0 |
| *Staurosirella martyi* (Heribaud) Morales & Manoylov | 1 | 0 | 0 | 0 | 0 | 1 | 0 |
| *Staurosirella pinnata* (Ehrenberg) Williams & Round | 1 | 0 | 0 | 0 | 0 | 1 | 0 |
| *Stephanodiscus hantzschi*i Grunow in Cleve & Grunow | 0 | 0 | 0 | 1 | 0 | 0 | 1 |
| *Stephanodiscus minutulus* (Kützing) Cleve & Moller | 0 | 0 | 0 | 1 | 0 | 1 | 0 |
| *Stephanodiscus parvus* Stoermer & Hakansson | 0 | 0 | 0 | 1 | 0 | 1 | 1 |
| *Surirella ovalis* Brébisson | 0 | 0 | 1 | 0 | 0 | 1 | 0 |
| *Tabularia fasciculata* (Agardh)Williams & Round | 1 | 0 | 0 | 0 | 1 | 1 | 1 |
| *Tabularia waernii* P.Snoeijs | 1 | 0 | 0 | 0 | 1 | 0 | 1 |
| *Thalassiosira baltica* (Grunow) Ostenfeld | 0 | 0 | 0 | 1 | 0 | 1 | 0 |
| *Thalassiosira levanderii* Van Goor | 0 | 0 | 0 | 1 | 0 | 1 | 0 |
| *Ulnaria ulna* (Nitzsch) Compére | 1 | 0 | 0 | 0 | 0 | 1 | 0 |
| unidentified | n/a | n/a | n/a | n/a | 1 | 1 | 1 |

**only in Puck Lagoon (Chałupy + Osłonino)**

| **name + authorities** | **High profile guild** | **Low profile guild** | **Motile guild** | **Planktonic** | **Chałupy** | **Osłonino** | **Gdynia** |
| --- | --- | --- | --- | --- | --- | --- | --- |
| *Achnanthes brevipes* var. *brevipes* Agardh | 1 | 0 | 0 | 0 | 1 | 1 | 0 |
| *Achnanthes delicatissima* Simonsen | 0 | 1 | 0 | 0 | 1 | 1 | 0 |
| *Amphora* sp. Ehrenberg | 0 | 1 | 0 | 0 | 1 | 1 | 0 |
| *Astartiella* sp. Witkowski, Lange-Bertalot & Metzeltin | 0 | 1 | 0 | 0 | 1 | 1 | 0 |
| *Bacillaria paxillifera* (O.F. Muller) Hendey var.paxillifera | 0 | 0 | 1 | 0 | 1 | 1 | 0 |
| *Ctenophora pulchella* (Ralfs ex Kützing) Williams & Round | 1 | 0 | 0 | 0 | 1 | 1 | 0 |
| *Diatoma tenuis* Agardh | 0 | 0 | 0 | 1 | 1 | 1 | 0 |
| *Diploneis didyma* (Ehrenberg) Ehrenberg | 0 | 0 | 1 | 0 | 1 | 1 | 0 |
| *Epithemia adnata* (Kutzing) Brebisson | 0 | 0 | 1 | 0 | 1 | 1 | 0 |
| *Fragilaria hyalina* (Kützing) Grunow | 1 | 0 | 0 | 0 | 1 | 1 | 0 |
| *Fragilaria improbula* Witkowski & Lange-Bertalot | 1 | 0 | 0 | 0 | 1 | 1 | 0 |
| *Fragilaria sopotensis* Witkowski & Lange-Bertalot | 1 | 0 | 0 | 0 | 1 | 1 | 0 |
| *Karayevia clevei* (Grunow) Bukhtiyarova | 0 | 1 | 0 | 0 | 1 | 1 | 0 |
| *Licmophora* C.Agardh | 1 | 0 | 0 | 0 | 1 | 1 | 0 |
| *Mastogloia pumila* (Grunow) Cleve | 0 | 0 | 1 | 0 | 1 | 1 | 0 |
| *Mastogloia smithii* Thwaites ex W.Smith | 0 | 0 | 1 | 0 | 1 | 1 | 0 |
| *Navicula hanseatica* Lange-Bertalot & Stachura | 0 | 0 | 1 | 0 | 1 | 1 | 0 |
| *Nitzschia capitellata* Hustedt in A.Schmidt & al. | 0 | 0 | 1 | 0 | 1 | 1 | 0 |
| Pennales | 0 | 0 | 1 | 0 | 1 | 1 | 0 |
| *Pseudostaurosira zeilleri* (Héribaud-Joseph) D.M.Williams & Round | 1 | 0 | 0 | 0 | 1 | 1 | 0 |
| *Skeletonema marinoi* Sarno & Zingone | 0 | 0 | 0 | 1 | 1 | 1 | 0 |
| sum | **7** | **4** | **8** |  |  |  |  |

**only in Gdynia station - Gulf of Gdańsk**

| **name + authorities** | **High profile guild** | **Low profile guild** | **Motile guild** | **Planktonic** | **Chałupy** | **Osłonino** | **Gdynia** |
| --- | --- | --- | --- | --- | --- | --- | --- |
| *Achnanthes* sp. J.B.M. Bory de St. Vincent | 0 | 1 | 0 | 0 | 0 | 0 | 1 |
| *Achnanthes* spec. G1 J.B.M. Bory de St. Vincent | 0 | 1 | 0 | 0 | 0 | 0 | 1 |
| *Amphora ovalis* (Kützing) Kützing | 0 | 1 | 0 | 0 | 0 | 0 | 1 |
| *Amphora* spec. G1 Ehrenberg | 0 | 0 | 1 | 0 | 0 | 0 | 1 |
| *Amphora* spec. G2 Ehrenberg | 0 | 0 | 1 | 0 | 0 | 0 | 1 |
| *Astartiella punctifera* (Hustedt) Witkowski & Lange-Bertalot | 0 | 1 | 0 | 0 | 0 | 0 | 1 |
| *Biremis lucens* (Hustedt) Sabbe, Witkowski & Vyverman | 0 | 1 | 0 | 0 | 0 | 0 | 1 |
| *Caloneis crassa* (Gregory) R.Ross | 0 | 0 | 1 | 0 | 0 | 0 | 1 |
| *Chaetoceros cf. fallax* Prosckina-Lavrenko | 0 | 0 | 0 | 1 | 0 | 0 | 1 |
| *Cocconeis hoffmannii* Simonsen | 0 | 1 | 0 | 0 | 0 | 0 | 1 |
| *Cocconeis irregularis* (P.Schulz) Witkowski | 0 | 1 | 0 | 0 | 0 | 0 | 1 |
| *Cocconeis* sp. Ehrenberg | 0 | 1 | 0 | 0 | 0 | 0 | 1 |
| *Cymbella* sp. Agardh | 1 | 0 | 0 | 0 | 0 | 0 | 1 |
| *Denticula subtilis* Grunow | 0 | 0 | 1 | 0 | 0 | 0 | 1 |
| *Epithemia* sp. Kützing | 0 | 0 | 1 | 0 | 0 | 0 | 1 |
| *Epithemia turgida* (Ehrenberg) Kützing var. *turgida* | 0 | 0 | 1 | 0 | 0 | 0 | 1 |
| *Fallacia* sp. Stickle et Mann | 0 | 0 | 1 | 0 | 0 | 0 | 1 |
| *Fistulifera saprophila* (Lange-Bertalot & Bonik) Lange-Bertalot | 0 | 0 | 1 | 0 | 0 | 0 | 1 |
| *Fragilaria* cf. *capensis* Grunow | 1 | 0 | 0 | 0 | 0 | 0 | 1 |
| *Grammatophora oceanica* Ehrenberg | 1 | 0 | 0 | 0 | 0 | 0 | 1 |
| *Halamphora acutiuscula* (Kützing) Levkov | 0 | 0 | 1 | 0 | 0 | 0 | 1 |
| *Halamphora luciae* (Cholnoky) Levkov | 0 | 0 | 1 | 0 | 0 | 0 | 1 |
| *Hippodonta arctica* (Patrick& Freese)Lange-Bertalot, Metzeltin & Witkowski | 0 | 0 | 1 | 0 | 0 | 0 | 1 |
| *Hippodonta hungarica* (Grunow) Lange-Bertalot Metzeltin & Witkowski | 0 | 0 | 1 | 0 | 0 | 0 | 1 |
| *Licmophora debilis* (Kützing) Grunow | 1 | 0 | 0 | 0 | 0 | 0 | 1 |
| *Navicula antonii* Lange-Bertalot | 0 | 0 | 1 | 0 | 0 | 0 | 1 |
| *Navicula cari* Ehrenberg | 0 | 0 | 1 | 0 | 0 | 0 | 1 |
| *Navicula* cf. *normaloides* Cholnoky | 0 | 0 | 1 | 0 | 0 | 0 | 1 |
| *Navicula* spp. Bory | 0 | 0 | 1 | 0 | 0 | 0 | 1 |
| *Navicula tripunctata* (O.F.Muller) Bory | 0 | 0 | 1 | 0 | 0 | 0 | 1 |
| *Navicula viminoides* spp. *cosmomarina* Lange-Bertalot, Witkowski, Bogaczewicz-Adamczak, Zgrundo | 0 | 0 | 1 | 0 | 0 | 0 | 1 |
| *Nitzschia hungarica* Grunow | 0 | 0 | 1 | 0 | 0 | 0 | 1 |
| *Nitzschia thermaloides* Hustedt | 0 | 0 | 1 | 0 | 0 | 0 | 1 |
| *Staurosira construens* Ehrenberg | 1 | 0 | 0 | 0 | 0 | 0 | 1 |
| *Stephanodiscus hantzschi*i Grunow in Cleve & Grunow | 0 | 0 | 0 | 1 | 0 | 0 | 1 |
| **sum** | **5** | **8** | **20** |  |  |  |  |
