# Supplementary material for: Mercury in the Diatoms of Various Ecological Formations
Source: Water Air Soil Pollut. 2018 May 11;229(5):168. doi: 10.1007/s11270-018-3814-1 (PMC5945792; doi:10.1007/s11270-018-3814-1)
Supplement: Supplementary file 3 — (DOCX 22 kb) [file 11270_2018_3814_MOESM3_ESM.docx]

**Electronic Supplementary Material 3**

Mercury in the photosynthetic microorganisms, Archives of Environmental Contamination and Toxicology, Magdalena Bełdowska*, Aleksandra Zgrundo, Justyna Kobos; ^*^corresponding author: [m.beldowska@ug.edu.pl](mailto:m.beldowska@ug.edu.pl), Institute of Oceanography, University of Gdańsk

Diatom taxa identified in phytoplankton samples.

Life form: p – planktonic, b – benthic; 1 – present; 0 - absent

| **Taxon + authorities** | **life form** | **Chałupy** | **Osłonino** | **Gdynia** |
| --- | --- | --- | --- | --- |
| *Achnanthes brevipes* var. *brevipes* Agardh | b | 1 | 0 | 0 |
| *Achnanthes* sp. J.B.M. Bory de St. Vincent | b | 1 | 0 | 0 |
| *Amphora hyalina* Kützing | b | 1 | 0 | 1 |
| *Amphora* sp. Ehrenberg | b | 1 | 0 | 1 |
| *Attheya decora* T.West | p | 1 | 0 | 0 |
| *Aulacoseira granulata* var. *granulata* (Ehrenberg) Simonsen | p | 1 | 0 | 1 |
| *Belonastrum berolinense* (Lemmermann) Round & Maidana | p | 0 | 1 | 0 |
| *Brebissonia lanceolata* (C.Agardh) R.K.Mahoney & Reimer | b | 1 | 0 | 0 |
| *Caloneis subsalina* (Donkin) Hendey | b | 0 | 1 | 0 |
| Centrales | p | 1 | 1 | 1 |
| *Chaetoceros* sp. Ehrenberg | p | 1 | 0 | 0 |
| *Chaetoceros wighamii* Brightwell | p | 1 | 1 | 1 |
| *Cocconeis pediculus* Ehrenberg | b | 0 | 1 | 1 |
| *Coscinodiscus granii* L.F.Gough | p | 1 | 1 | 0 |
| *Coscinodiscus* sp. Ehrenberg | p | 1 | 1 | 0 |
| *Ctenophora pulchella* (Ralfs ex Kützing) Williams & Round | b | 1 | 1 | 1 |
| *Cyclotella atomus* Hustedt | p | 1 | 1 | 1 |
| *Cyclotella choctawhatcheeana* Prasad | p | 1 | 0 | 1 |
| *Cyclotella meneghiniana* Kützing | p | 0 | 1 | 0 |
| *Cyclotella* sp. (Kützing) Brébisson | p | 1 | 0 | 0 |
| *Cylindrotheca closterium* (Ehrenberg) Reimann & J.C.Lewin | p/b | 1 | 0 | 1 |
| *Dactyliosolen fragilissimus* (Bergon) Hasle | p | 1 | 0 | 0 |
| *Diatoma tenuis* Agardh | p | 0 | 1 | 0 |
| *Diploneis didyma* (Ehrenberg) Ehrenberg | b | 1 | 1 | 1 |
| *Diploneis elliptica* (Kützing) Cleve | b | 1 | 0 | 0 |
| *Entomoneis paludosa* (W.Smith) Reimer | b | 1 | 0 | 0 |
| *Epithemia* sp. Kützing | b | 1 | 1 | 1 |
| *Epithemia turgida* (Ehrenberg) Kützing | b | 1 | 0 | 0 |
| *Fragilaria crotonensis* Kitton | p | 1 | 0 | 1 |
| *Fragilaria* Lyngbye | p/b | 1 | 1 | 1 |
| *Gomphonema* cf. *olivaceum* var. *balticum* (Cleve) Grunow | b | 1 | 1 | 1 |
| *Gomphonema* sp. Ehrenberg | b | 0 | 0 | 1 |
| *Grammatophora marina* (Lyngbye) Kützing | b | 1 | 1 | 1 |
| *Halamphora coffeaeformi*s (C.Agardh) Levkov | b | 1 | 0 | 0 |
| *Licmophora* sp. C.Agardh | b | 1 | 1 | 1 |
| *Melosira* cf. *arctica* Dickie | p | 1 | 1 | 1 |
| *Melosira moniliformis* (O.F.Müller) C.Agardh | p | 0 | 0 | 1 |
| *Melosira varians* C.Agardh | p | 0 | 1 | 0 |
| *Meridion circulare* (Greville) C.Agardh | p | 0 | 1 | 1 |
| *Navicula* spp. Bory | p/b | 1 | 1 | 1 |
| *Nitzschia longissima* (Brébisson) Ralfs | p/b | 1 | 0 | 0 |
| *Nitzschia* sp. Hassall | p/b | 1 | 1 | 1 |
| *Pauliella taeniata* (Grunow) F.E.Round & Basson | p | 1 | 1 | 1 |
| Pennales | p/b | 1 | 1 | 1 |
| *Pleurosigma formosum* W.Smith | p/b | 1 | 0 | 1 |
| *Pleurosigma subsalsum* Wislouch & Kolbe | b | 1 | 1 | 0 |
| *Rhoicosphenia abbreviata* (C.Agardh) Lange-Bertalot | b | 1 | 1 | 0 |
| *Skeletonema marinoi* Sarno & Zingone | p | 1 | 0 | 1 |
| *Stephanodiscus hantzschi*i Grunow in Cleve & Grunow | p | 1 | 1 | 1 |
| *Surirella* sp. Turpin | b | 0 | 0 | 1 |
| *Tabularia fasciculata* (Agardh)Williams & Round | b | 1 | 1 | 1 |
